# Indents

**Published:** 1918-01

**Authors:** 


### INDENTS

☞ **Brief Articles of Technical or Scientific Value will receive notice and comment. Contributions invited.**

*To Lessen Pain* After an extraction, where bone has been removed by burs or chisels, or where alveolar process is exposed, dry the surface and apply ninety-five per cent. phenol, then neutralize with alcohol. This will lessen the post-operative pain. —R. E. Light, *Dental Review.*

*Salt Solution as a Neutralizing Agent* Recently my nurse, in handing me a bottle of silver nitrate, saturated solution, spilled a considerable portion of it in my hands. In order to neutralize the cauterizing effect, I washed my hands in a saturated salt solution. I was much surprised to find that my hands did not discolor. Try it next time.—Dr. W. M. Wadleigh, *Pacific Gazette.*

*Medical Fallacies* A fallacy of the present theory of medicine is to attribute many morbid conditions to the mouth and teeth and resort to their extraction on suspicion. There is no question in my mind as to the fallacy of depriving people of their teeth and robbing them of their efficiency. It is a great shock to the physical economy, and will not assist in removing the infection. The great cause of morbid conditions is in the gastro-intestinal tract, and in improper diet.—H. E. Bliler, D.D.S., *Health Culture.*

*Somewhat Put Out* The latest example of English as she is spoken comes from Egypt, where a native interpreter, who had overstayed his leave, wrote the following letter to his chief:

"My absence is impossible. Some one has removed my wife. My God, I am annoyed."—*New York Sun.*

*United States Dental School in Philadelphia* Announcement is made that the Surgeon-General has ordered the establishment in Philadelphia of a school for training in oral surgery and plastic reconstruction of the face and jaws of men who have been maimed in the war. Twenty-five or thirty recently appointed officers of the medical reserve service will be detailed for didactic and laboratory study at the institution. Dr. Wilmer Krusen, director of public health and charities, has agreed to afford facilities in the dental wards of the Philadelphia General Hospital for clinical demonstrations, and has offered his office in city hall for meetings of the teaching board. Dr. Charles R. Turner, dean of the Evans Institute of Dental Surgery, will have charge of the organization of the teaching staff. He will be assisted by Dr. Herman Prinz of the institute in selecting the staff. Lectures and operators from other medical and dental schools of Philadelphia, who are specialists in oral surgery, will become members of the staff.

*The Ash on the Cigar* Young men, very young men, in their earlier smoking days like to keep the ash on a cigar as long as it will stay. Somebody has told them that it is a mark of a good cigar for it to have an ash that does not crumble and fall off; it is at least true that a long ash marks the cigar as one made with a long filler.

The young man smoker who likes to see the ash grow and hang on handles his cigar with great care so as not to joggle it off, and if he smokes up the whole cigar without dropping the ash he thinks that he has been smoking a good cigar and that he is a good smoker.

As he grows older the young man comes to be a smoker of another sort. He flicks the ashes off his cigar jauntily. Then as he grows older still he may come to be careless about the ash, or he may be so engrossed with other things as he smokes that he lets the ash accumulate, to drop maybe on his garments and cover them with ashes.

Still later in life the grown, mature man is likely to knock the ash off his cigar at frequent intervals, to keep it down short. He smokes in a business-like way, he doesn't want the ashes scattered over him. But as he grows older yet, when he comes to be an oldish man, he may come again as he smokes to keep the ash on a cigar as long as he can.

You may see an old man smoking a cigar thus, holding it carefully and smoking slowly and contemplatively, looking at the ash occasionally and clearly interested in seeing it grow, just as he did when he was young. Why he does this now is uncertain, but perhaps it is because it brings back memories of his youth.

*A Logical Nomenclature* "So you have twins at your house?" said Mrs. Nabor to little Jack. "Yessum," he said soberly, "two of them." "What are they going to call them, my dear?" "Well, I don't know for sure, but I think their names is Thunder and Lightnin', 'cause that's the names Papa called them when the doctor came in and told him about them."

*Quiet and Comfort* I have a longing to experience the stillness of undisturbed nature and be free from the noises made by man. Those that are produced by wind and sea, and others of nature, do not disturb me. I like all of them.

I like, too, to see the flash of lightning and listen to the roll of thunder, they cause me exhilaration, never fear. I can sleep without waking even with the heaviest thunder taking place.

The noise of a rainstorm does not waken me at night. Oh, glorious nature! The great drama of the earth and skies, open to all, nothing to pay, that is the entertainment for me.—Dr. Lee in *Health Culture.*

